# Zygosity and autonomic stress reactivity during social exclusion: biofeedback findings from the TwinCord-EDID study

**DOI:** 10.3389/fpsyt.2026.1865772

**Published:** 2026-06-24

**Authors:** Anna Zalpur, Nazar Mazurak, Sophia Kristina Wolf, Katja Weimer, Jeannette Hübener-Schmid, Miriam Goebel-Stengel, Stephan Zipfel, Andreas Stengel

**Affiliations:** 1 Department of Psychosomatic Medicine and Psychotherapy, University Hospital Tübingen, Tübingen, Germany; 2 German Center for Mental Health (DZPG), site Tübingen, Tübingen, Germany; 3 Clinic for Psychosomatic Medicine and Psychotherapy, Klinikum Stuttgart, Stuttgart, Germany; 4 Clinic for Internal Medicine, Diakonissen-Stiftungs-Krankenhaus Speyer, Speyer, Germany; 5 Department of Psychosomatic Medicine and Psychotherapy, Ulm University Medical Center, Ulm, Germany; 6 Institute of Medical Genetics and Applied Genomics, University of Tübingen, Tübingen, Germany; 7 Falculty Life Science, Reutlingen University, Reutlingen, Germany; 8 Clinic for Internal Medicine, SRH Clinic Sigmaringen, Sigmaringen, Germany

**Keywords:** biofeedback, cyberball paradigm, eating disorder, gastrointestinal disorder, twin study

## Abstract

**Objective:**

Patients with functional gastrointestinal disorders (FGID) and/or eating disorders (ED) often report increased sensitivity to psychosocial stress. This exploratory study examined autonomic responses, measured via heart rate variability (HRV) and biofeedback, to experimentally induced social exclusion (Cyberball task) in monozygotic and dizygotic twins with and without questionnaire-based symptoms symptoms for FGID and ED. The twin design was used to explore potential genetic and environmental contributions to stress reactivity.

**Methods:**

35 monozygotic and dizygotic twin pairs participated. Zygosity was determined via genetic blood testing. Participants were assigned to four groups based on questionnaire data assessing symptoms for ED and/or FGID: (I) ED-related symptoms, (II) FGID-related symptoms, (III) combined symptoms, and (VI) asymptomatic controls. All participants completed a controlled social exclusion task using the Cyberball paradigm. Heart rate variability (HRV), skin conductance (SC), skin temperature (ST) and subjective stress ratings (VAS) were assessed during baseline, exclusion and recovery phases. Analyses focused on exploratory group comparisons as well as twin-based concordance, intraclass correlations and Falconer estimates. The study was approved by the Ethics Commission at the Medical Faculty of Eberhard-Karls-Universität and at Tübingen University Hospital (project number 094/2022B01). All subjects gave written informed consent.

**Results:**

Across the total sample, SC, ST, HRV, and VAS significantly changed across experimental phases, confirming successful stress induction and recovery. No significant differences emerged between symptom groups. Exploratory analyses revealed zygosity-related interactions for selected HRV indices and birth-order effects in VAS. Twin-based analyses suggested that non-shared environmental factors may contribute substantially to variability in physiological stress response, whereas possible genetic influences were observed for subjective stress ratings and selected HRV indices. However, these findings should be interpreted cautiously given the limited sample size and exploratory nature of the analyses.

**Conclusion:**

Social exclusion induced clear autonomic and subjective stress responses across all participants, independent of symptom status. While no group differences were observed, exploratory twin analyses provide preliminary indications that both environmental and genetic factors may contribute to individual differences in stress reactivity. Dute to the sample size and the exploratory design, these findings should be regarded as hypotheses-generating and require replication in larger twin cohorts.

## Introduction

Eating disorders (e.g. anorexia nervosa, bulimia nervosa, binge eating disorder) and functional gastrointestinal disorders (e.g. irritable bowel syndrome) frequently co-occur together and may persist even after recovering from one of the various types of eating disorders (1–3). Patients with ED and/or FGID appear to respond differently to stressful situations (4, 5), showing altered autonomic nervous system activity in response to stress-related stimuli (3, 6, 7) and even a different emotion regulation (8). In particular, differences in heart rate variability have been consistently reported across several studies before (9–11). One potential trigger that may exacerbate the symptoms for ED or FGID is social exclusion, which may act not only as a precipitating factor but also as a consequence of these conditions (12, 13). While altered autonomic nervous system responses have been observed in both conditions, the relative contribution of genetic and environmental factors to these responses remains poorly understood.

To better characterize these autonomic stress responses and to create a basis for disentangling genetic and environmental influences, experimental paradigms that allow controlled stress induction together with physiological assessment are required. Biofeedback techniques – particularly heart rate variability (HRV), as well as skin conductance (SC) and skin temperature (ST) – have been employed in various studies as therapeutic tools to help patients regulate physiological responses (14–17). The cyberball paradigm enables to stimulate controlled social exclusion while continuously recording biofeedback data and therefore assessing participants’ autonomic nervous system reactivity (18, 19). HRV served as a marker of vagal activity and parasympathetic activation (20–22). SC, also referred to as electrodermal activity (EDA), reflects the activity of the sweat glands which are regulated by the sympathetic nervous system (23). SC typically increases during emotional arousal, stress situations or perceived social threats (24). Distal skin temperature, measured via finger sensors, indicates peripheral blood circulation (25). During stress responses, ST generally decreases due to peripheral vasoconstriction; a delayed return to baseline may indicate impaired autonomic recovery (26, 27).

In addition to physiological indicators, perceived stress can also be assessed using a visual analogue scale (VAS), which allows to capture both objective, but also subjective responses during social exclusion (28). While biofeedback measures such as HRV, SC and ST allow detailed tracking of stress physiology, the origins of inter-individual variability in these responses are less clear. A twin design provides a unique framework to assess genetic and environmental influences on stress reactivity.

While numerous studies have addressed the physiological stress responses in individuals with ED and FGID (29, 30), relatively little is known about how genetic predisposition versus environmental exposure influences these responses. Twin studies offer a unique opportunity to investigate such interactions, enabling researchers to differ between heritable and non-heritable contributors to the origin of different diseases (31–33). While monozygotic (MZ) twins share almost 100% of their genetic material, dizygotic (DZ) twins share on average approximately 50% with their sibling (34, 35). When twins are raised together, environmental influences are also largely comparable, allowing researchers to assess the relative contributions of genetics and environment, to analyze the possible disease triggers. If the condition shows a higher rate of concordance within the MZ twin pairs compared to the DZ twins, the genetic influence might play a bigger role than the environmental factor (36). Conversely, differences in response within MZ twin pairs, despite their identical genetic overlap, point toward environmental influences. Twin studies are particularly well-suited for the purpose of investigating the influence of genetic and environment, as intra-class correlations (ICC) and heritability estimates (Falconer’s formula) can be used to quantify the impact of them to physiological and psychological stress responses (37, 38).

Previous twin studies have demonstrated that genetic factors contribute to the development of functional gastrointestinal disorders (39, 40). However, it remains unclear how the impact of genetic might affect the therapeutic response, such as physiological reactions to biofeedback interventions. Our study aims to address this gap by comparing autonomic reactivity in monozygotic and dizygotic twins with varying symptom profiles during a standardized social exclusion task. To our knowledge, no study has combined a twin comparison with a controlled social stress paradigm and real-time physiological monitoring to examine stress reactivity in participants with questionnaire-based symptoms for ED and/or FGID.

Given the high comorbidity of ED and FGID and the central role of stress sensitivity in both, understanding autonomic responses to social exclusion is of particular relevance in psychosomatic medicine (41, 42). The present study aimed to explore autonomic stress reactivity during experimentally induced social exclusion in monozygotic (MZ) and dizygotic (DZ) twins with and without questionnaire-based symptoms suggestive of ED and/or FGID. We further explored whether zygosity influenced concordance in physiological and subjective stress responses and whether birth order contributes to differential stress regulation within twin pairs. Given the relatively small sample size and exploratory design, analyses focused on descriptive patterns, intra-class correlations and exploratory heritability estimates rather than confirmatory testing. Accordingly, findings regarding genetic and environmental influences should be regarded as preliminary and hypothesis-generating.

## Materials and methods

### Study design

This study was designed as a prospective observational twin study with an experimental social stress induction component using the cyberball paradigm. Data collection occurred over a one-year period. All assessments were conducted between 12 pm and 6 pm. The measurements for each twin pair were assessed on the same day, with measurements taken consecutively. Given the exploratory nature of the study and the limited sample size, analyses focused on descriptive group comparisons as well as exploratory twin-based analyses including intraclass correlations (ICC) and Falconer estimates.

### Participants and procedure

The participants are monozygotic and dizygotic twin pairs that are part of the TwinHealth Registry in Tübingen (32), a project of the TwinHealth consortium at the Faculty of Medicine of the University of Tübingen. The registry, established in 2016, was designed to recruit twin pairs interested in contributing to research on various scientific and medical questions. Currently, the TwinHealth cohort includes over 600 twin pairs who provided written informed consent for data collection and potential participation in studies such as the present one. The study was approved by the Ethics Commission at the Medical Faculty of Eberhard-Karls-Universität and at Tübingen University Hospital (project number 094/2022B01). All subjects involved in the study gave their written consent.

A total of 35 twin pairs were assessed during the study period, including 21 monozygotic and 14 dizygotic pairs. Inclusion criteria required the participation of both twins and the presence of both during the day of measurement as well as sufficient proficiency in German to complete all study tasks. Included participants ranged in age from 33 to 78.

Before taking part in the study, participants completed a questionnaire about assessing eating behavior, gastrointestinal symptoms, and emotional responses related to food. Eating disorder-related symptoms were assessed using the Eating Attitudes Test (EAT-40), while gastrointestinal symptoms were assessed using the Gastrointestinal Symptom Rating Scale (GSRS-15). For the EAT-40, items 1, 18, 19, 23 and 27 were reverse-coded according to the scoring instructions. A total score ≥ 15 was considered indicative of clinically relevant eating-disorder-related symptomatology. For the GSRS-15, the total score was divided by the number of items, resulting in a mean score ranking from 1 to 7. A mean score ≥ 1.5 was used as the threshold for FGID-related symptoms.

Based on questionnaire screening results, twin pairs were assigned to one of four symptom groups:

- twins with eating disorder-related symptoms only (EAT).
- pairs with functional gastrointestinal disorder-related symptoms only (FGID).
- twins with both eating disorder-related and FGID-related symptoms (EAT+FGID).
- asymptomatic twin pairs (ASYMP).

Twin pairs were classified at the pair level. Pairs were assigned to the EAT, FGID or EAT+FGID group if at least one twin fulfilled the respective screening criteria. Pairs in which neither twin met screening criteria were assigned to the ASYMP group. These classifications were based on questionnaire screening and do not represent formal clinical diagnoses.

Within the symptom groups, twin pairs were further categorized as concordant or discordant. Concordant pairs were defined as both twins fulfilling the respective screening criteria. Discordant pairs were defined as cases in which one twin fulfilled screening criteria for eating disorder-related symptoms, FGID-related symptoms or both, while the co-twin did not meet screening criteria for either conditionVariables such as BMI, medication use, smoking status, caffeine consumption, menstrual cycle phase and physical activity were not systematically assessed and therefore could not be included in the analyses.

### Zygosity determination

Zygosity was determined via genetic testing. Blood samples were collected from each twin pair on the day of participation and analysed at the Institute of Human Genetics at the University of Tübingen. Based on genetic analysis, zygosity was classified as monozygotic or dizygotic. Zygosity results were communicated to participants after completion of the study.

### Measures

To assess the heart rate variability (HRV), we used the Nexus 10 MKII biofeedback system from MindMedia (12). HRV, distal skin temperature and skin conductance were recorded continuously using three finger sensors attached to the distal phalanges of the participant’s right hand.

The measurement procedure was divided into three phases:

1. Baseline phase: participants sat quietly in a relaxed seated position for a 5-minute baseline recording.
2. Cyberball task: twin pairs completed the Cyberball task on a computer screen positioned in front of them. The task included two phases:
  • Inclusion phase: the participant believed they were engaged in a virtual ball-tossing game with two other players online.
  • Exclusion phase: after approximately 1.5 to 2 minutes (depending on individual reaction speed), the participant was progressively excluded from the game. The exclusion lasted for about 1.5 minutes until the game was finished.
3. Recovery phase: after the task, the twins were asked to remain still and silent for an additional 5-minute recovery recording.

The participants did not know about the experimental exclusion, nor did they know that their two co-players were not actually online playing with them (43, 44). To evaluate the subjective change of stress perception (45), we asked the participants to classify themselves on a virtual rating scale (VRS). We assessed their answers on a scale from 1 to 10, 1 being “very relaxed” and 10 being “very stressed”. Ratings were assessed at four time points: first before baseline measurement, second after the baseline phase, third immediately following the Cyberball task and lastly after the recovery phase (46).

All physiological and subjective measures were later entered into twin analyses, including ICC and heritability estimates.

Within each twin pair, the first-born twin was always assessed before the second-born twin. Birth order was determined based on participant self-report.

### Data analysis

To analyze and process the collected data we used the BioTrace+ Software for NeXus-10 (Version 2018A1, MindMedia) and the Kubios HRV Premium 3.4.1 programme and exported the edited data into an excel table (47). The final analyses and statistics were performed using IBM SPSS Statistics 29.0.1.1 and Microsoft Excel.

For skin conductance (SC) and skin temperature (ST), recordings were exported from BioTrace+ Software at a sampling rate of 32 sampes per second (32 SPS) using tab-delimited ASCII-files. Mean values were calculated separately for the four predefined experimental phases:

1. Baseline: beginning of recording until Marker 1.
2. Inclusion: Marker 1 to Marker 2.
3. Exclusion: Marker 2 to Marker 3.
4. Recovery: Marker 3 until the end of recording.

For each participant, mean SC and ST values were calculated for all four phases and subsequently entered into the statistical database.

For heart rate variability (HRV) analyse, recordings were exported from BioTrace+ Software in EDF format and imported into Kubios HRV Premium 3.4.1.

HRV parameters were analyzed separately for baseline, inclusion, exclusion and recovery phases. While baseline and recovery recordings lasted five minutes and inclusion and exclusion phases approximately two minutes each, a standardized two-minute analysis window was used for all HRV calculations to ensure comparability across phases. No additional artefact correction procedures beyond the standard Kubios processing workflow were applied.

The following parameters were extracted for statistical analyses: MeanRR (ms), SDNN (ms), RMSSD (ms), LFpow_AR (ms²), HFpow_AR (ms²), LFpow_AR (log), HFpow_AR (log), LFpow_AR (n.u.), HFpow_AR (n.u.), and LF/HF ratio.

Subjective stress perception was assessed using a visual rating scale ranging from 1 (“very relaxed”) to 10 (“very stressed”). Ratings obtained at the four assessment time points were entered into the statistical database and analyzed analogously to the physiological parameters.

Repeated-measures mixed analyses of variance (mixed ANOVA) were conducted to assess phase-related changes in physiological and subjective stress responses across symptom groups. Additional analyses examined interactions involving zygosity (monozygotic vs. dizygotic) and birth order (first-born vs. second-born twin). Significant effects were further explored using *post-hoc* comparisons where appropriate.

The main focus of the study was the investigation of within-pair concordance and zygosity-related differences, while also examining group-related effects. Intraclass correlations (ICC) were calculated separately for monozygotic and dizygotic twin pairs to assess within-pair similarity in stress responses. Falconer’s formula was subsequently applied to estimate the relative contributions of additive genetic factors (h^2^), shared environmental factors (c^2^) and unique environmental factors (e^2^).

Given the exploratory nature of the study, no correction for multiple comparisons was applied. Findings should therefore be interpreted with caution and regarded as hypothesis-generating rather than confirmatory, particularly with respect to ICC- and Falconer-based estimates.

## Results

### Sample characteristics

A total of 35 twin pairs participated in the study (21 monozygotic, MZ); 14 dizygotic, DZ). Based on symptom profiles, nine pairs were asymptomatic (ASYMP), seven pairs reported questionnaire-based symptoms of eating disorders (EAT), thirteen pairs reported functional gastrointestinal disorders-related symptoms (FGID) and six pairs presented with questionnaire-based symptoms of both ED and FGID (EAT+ FGID).

Overall, 19 pairs (54.3%) were concordant for group membership, whereas 16 pairs (45.7%) were discordant. Concordance was more frequent in MZ twins (61.9%) than in DZ twins (42.9%). All asymptomatic pairs were concordant (100%), while the questionnaire-based symptom groups showed varying proportions of concordance and discordance (**Table 1**).

**Table 1:** Concordance and discordance rates among monozygotic and dizygotic twin pairs by symptom group.

Group	Pairs (=n)	Concordant total	Discordance total	Concordance MZ n (%)	Discordance MZ n (%)	Concordance DZ n (%)	Discordance DZ n(%)
EAT	7	2 (28.6%)	5 (71.4%)	2 (40.0%)	3 (60.0%)	0 (0.0%)	2 (100%)
EAT+FGID	6	3 (50.0%)	3 (50.0%)	1 (33.3%)	2 (66.7%)	2 (66.7%)	1 (33.3%)
FGID	13	5 (38.5%)	8 (61.5%)	4 (57.1%)	3 (42.9%)	1 (16.7%)	5 (83.3%)
ASYMP	9	9 (100%)	0 (0.0%)	6 (100%)	-	3 (100%)	-
Total	35	19 (54.3%)	16 (45.7%)	13 (61.9%)	8 (38.1%)	6 (42.9%)	8 (57.1%)

### Skin temperature

**Figure 1:**
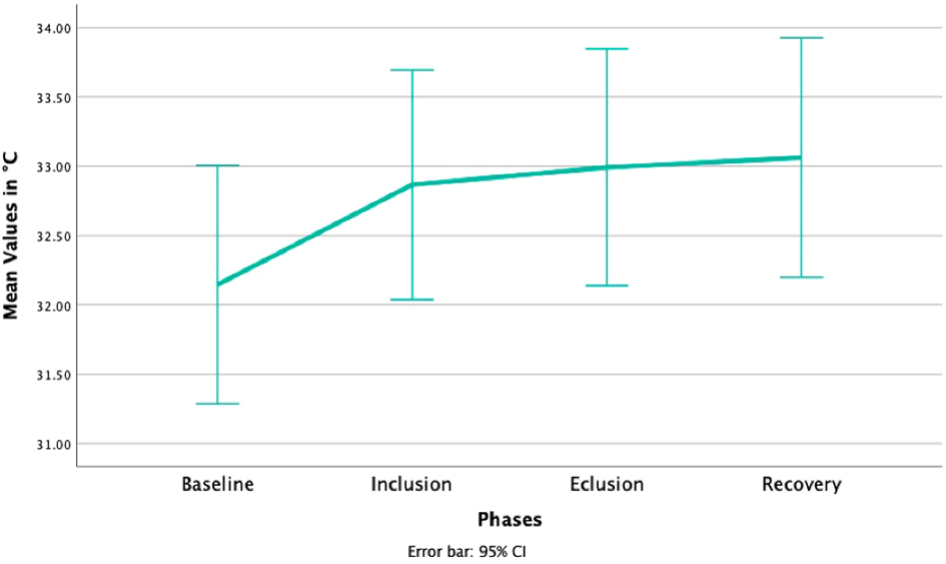
Mean skin temperatures (in °C) across the four phases of the Cyberball task (baseline, inclusion, exclusion, recovery). Error bars = standard error of the mean.

Skin temperature (ST) also varied significantly across phases (p < 0.001), showing increases starting at the beginning of the Cyberball-task (**Table 2**; **Figure 1**). No significant group effect was observed (p= 0.243) and no interaction between phase and group emerged. *Post-hoc* tests confirmed no significant group differences (all p > 0.05).

**Table 2:** Mean skin temperature values (°C) across cyberball phases in the different symptom groups.

Phase	EAT	EAT+FGID	FGID	ASYMP
Baseline	31.05 ± 4.58	32.79 ± 2.92	32.34 ± 3.63	32.28 ± 3.23
Inclusion	31.20 ± 4.57	33.67 ± 1.97	33.37 ± 3.37	32.89 ± 3.26
Exclusion	30.97 ± 4.74	33.78 ± 2.01	33.51 ± 3.52	33.29 ± 3.12
Recovery	31.23 ± 4.75	33.85 ± 3.54	33.60 ± 3.54	33.19 ± 3.30

Values are shown as mean values ± standard deviations in °C (degree Celsius).

### Skin conductance

**Figure 2:**
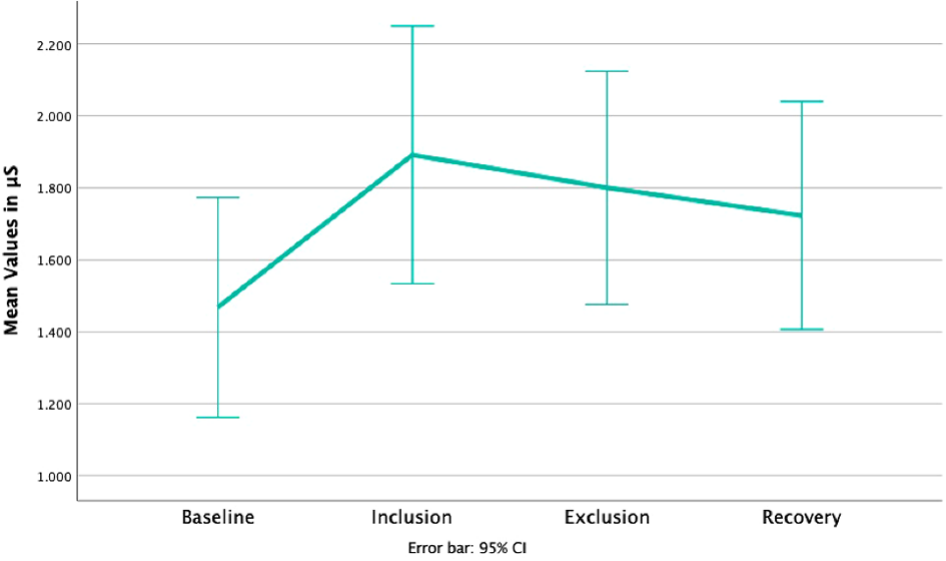
Mean values of skin conductance (in µS) across the four phases of the Cyberball task (baseline, inclusion, exclusion, recovery). Error bars = standard error of the mean.

Skin conductance (SC) changed significantly across phases (F = 39.33, p < 0.001) with higher values during the Cyberball task compared to baseline and recovery (**Figure 2**, **Table 3**). No significant main effect of group was found (F = 1.09, p = 0.358) and there was no significant interaction between phase and group (p = 0.25). *Post-hoc* comparisons confirmed no significant between-group differences (all p > 0.50).

**Table 3:** Mean skin conductance across experimental phases by symptom group.

Phase	EAT	EAT+FGID	FGID	ASYMP
Baseline	1.8 ± 1.17	1.22 ± 0.48	1.48 ± 1.41	1.84 ± 1.52
Inclusion	1.46 ± 1.39	1.68 ± 0.74	1.89 ± 1.66	2.38 ± 1.68
Exclusion	1.44 ± 1.35	1.71 ± 0.89	1.75 ± 1.36	2.22 ± 1.59
Recovery	1.28 ± 1.18	1.57 ± 0.79	1.68 ± 1.24	2.23 ± 1.72

Values are shown as mean values ± standard deviations in µS (microsiemens).

### Heart rate variability

Several HRV indices changed significantly across phases of the Cyberball task (**Table 4**). The standard deviation of NN intervals (SDNN) was higher during the exclusion phase compared to other phases. Low frequency power (LF) also showed significant phase effects. Similarly, high frequency power in normalized units (HFpow_AR(n.u.)) varied across phases. The mean NN interval (MeanRR), the root mean square of successive differences (RMSSD), high-frequency power (HFpow_AR-ms^2^, log-transformed) and the low-frequency to high frequency ratio (LF/HF) ratio remained stable (all p > 0.05).

**Table 4:** Results of variance analyses for heart rate variability parameters during Cyberball task.

Parameter	Main effect phase	Main effect group	Phase x group	Phase x zygosity	Phase x birth order
Mean_RR (ms)	F= 3.047, p= 0.077	F= 0.743, p= 0.141	F= 0.150, p= 0.956	F= 0.260, p= 0.707	F= 0.313, p= 0.628
SDNN (ms)	F= 3.028, p= 0.039*	F= 0.869, p= 0.463	F= 1.124, p= 0.351	F= 0.700, p= 0.534	F= 0.548, p= 0.624
RMSSD (ms)	F= 1.983, p= 0.144	F= 1.390, p= 0.256	F= 0.976, p= 0.444	F= 0.227, p= 0.791	F= 1.118, p= 0.330
LFpow_ AR (ms^2^)	F= 1.989, p= 0.135	F= 0.603, p= 0.616	F= 0.487, p= 0.838	F= 0.388, p= 0.704	F= 0.646, p= 0.544
HFpow_ AR (ms^2^)	F= 0.192, p= 0.155	F= 1.592, p= 0.203	F= 0.993, p= 0.425	F= 0.114, p= 0.845	F= 3.066, p= 0.064
LFpow_ AR (log)	F= 5.083, p= 0.002*	F= 1.483, p= 0.230	F= 1.831, p= 0.067	F= 0.584, p= 0.626	F= 0.499, p= 0.684
HFpow_ AR (log)	F= 2.497, p= 0.062	F= 1.315, p= 0.279	F= 0.754, p= 0.659	F= 0.078, p= 0.972	F= 1.236, p= 0.299
LFpow_ AR (n.u.)	F= 2.872, p= 0.049*	F= 1.455, p= 0.237	F= 1.684, p= 0.114	F= 0.394, p= 0.718	F= 1.888, p= 0.146
HFpow_ AR (n.u.)	F= 2.876, p= 0.049*	F= 1.453, p= 0.238	F= 1.681, p= 0.115	F= 0.395, p= 0.718	F= 1.890, p= 0.145
LF_HF_ ratio	F= 0.328, p= 0.749	F= 0.326, p= 0.806	F= 1.748, p= 0.106	F= 0.818, p= 0.458	F= 0.450, p= 0.665

The results of the variance analyses for the heart rate variability parameters during the Cyberball task are shown. The F and p values are given for the main effect phase (baseline, inclusion, exclusion, recovery), group (EAT, EAT+FGID, FGID, ASYMP) and their interactions with zygosity (monozygotic/dizygotic) and birth order (first/second born). Significance assumed at p<0.05, marked with *.

No significant main effects of group were detected for HRV indices (all p > 0.05) and interactions between phase and group were not significant. However, interaction effects involving zygosity were observed. RMSSD showed a significant group x zygosity interaction (F = 2.87, p= 0.045) as did HFpow_AR (ms^2^) (F = 4.875, p = 0.005) and HFpow_AR (log) (F = 5.064, p = 0.004). The birth order did not show any significant influence.

### Subjective stress ratings

**Figure 3:**
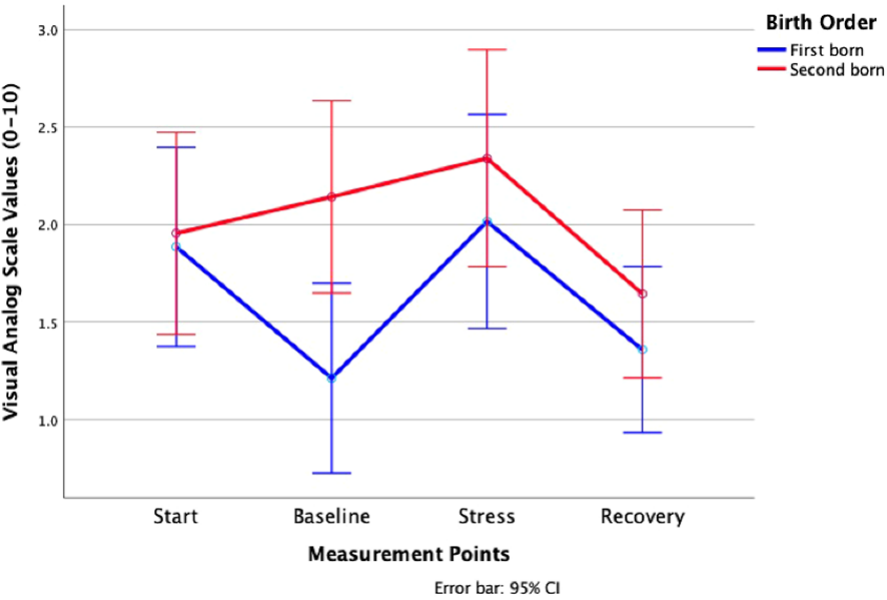
Course of the mean values of the visual analogue scale (0-10) separated by birth order over the four measurement points. Error bars = 95% confidence interval.

Subjective stress ratings (VAS) changed significantly across phases (F = 8.05, p < 0.001). Ratings increased from baseline to exclusion and decreased again during recovery (**Table 5**). There were no significant effects of group (F = 0.83, p = 0.590) or zygosity (F = 0.93, p = 0.428). However, birth order showed a significant effect on within-subject changes across phases (F = 3.16), p = 0.026) with first- and second born twins reporting different subjective stress ratings across the experimental phases (**Figure 3**).

**Table 5:** Subjective stress ratings during the experimental phases.

Phase	EAT	EAT+FGID	FGID	ASYMP
Start	1.14 ± 1.23	2.08 ± 1.44	2.38 ± 1.44	2.11 ± 1.75
Baseline	0.50 ± 0.86	2.08 ± 1.44	1.92 ± 1.52	2.00 ± 1.50
Stress	1.14 ± 1.10	2.42 ± 1.17	2.46 ± 1.24	2.67 ± 2.11
Recovery	0.50 ± 0.65	1.67 ± 0.89	1.62 ± 1.17	2.11 ± 1.26

Mean values ± standard deviations of subjective tension (VAS; 0= relaxation, 10= tension) at the four measurement points (start, baseline, stress, recovery) separated according to the four groups (EAT, EAT+FGID, FGID, ASYMP).

### Twin analyses

*Concordance and discordance rates:* As noted above, MZ pairs were more often concordant (61.9%) than DZ pairs (42.9%). The asymptomatic group showed complete concordance (100%). Symptom groups showed mixed patterns with higher discordance particularly in the EAT and FGID groups.

*Intra-class correlations (ICC) and Falconer estimates:* For skin temperature, ICCs were not significant (MZ: F = 1.102, p = 0.415; DZ: F = 1.036, p = 0.475). According to exploratory Falconer estimates h^2^ = 5.4%, c^2^ = 0% and e^2^ = 95.5%.

The skin conductance showed no significant ICCs (MZ: F = 0,495, p = 0.938; DZ: F = 1,177, p = 0.387). Exploratory Falconer estimates,h^2^ = 0%, c^2^ = 17.4%) and e^2^ was 100%.

For subjective stress ratings, MZ twins showed a significant ICC (F = 2.242, p = 0.039), whereas DZ twins did not (F = 0.904, p = 0.571). Exploratory Falconer estimates h^2^ = 75.6%, c^2^ = 0%, e^2^ = 62.2%.

For HRV, MeanRR showed a significant ICC among MZ twins (F = 6.295, p < 0.001), but not among DZ twins (F = 0.610, p = 0.808). Exploratory Falconer estimates h² = 144.6%, c^2^ = 0%, e² = 27.7%. For SDNN, RMSSD, LF, and HF parameters, no significant ICCs were found. Corresponding exploratory Falconer estimates for these variables are presented in **Table 6**.

**Table 6:** Intraclass correlations and Falconer formulas.

Variable	ICC_MZ	ICC_DZ	h^2^	c^2^	e^2^
HLF_react	-0.324 = 0	0.087	-0.174 = 0%	0.174 = 17.4%	1 = 100%
Temp_react	0.045	0.018	0.054 = 5.4%	-0.009 = 0%	0.955 = 95.5%
VRS_react	0.378	0.038 = 0	0.756 = 75.6%	-0.378 = 0%	0.622 = 62.2%
MeanRR_react	0.723	-0.202 = 0	1.446 = 144.6%	-0.723 = 0%	0.277 = 27.7%
SDNN_react	-0.183 = 0	0.061	-0.122 = 0%	0.122 = 12.2%	1 = 100%
RMSSD_react	-0.079 = 0	0.161	-0.322 = 0%	0.322 = 32.2%	1 = 100%
LFpow_ARms2_react	0.339	-0.231 = 0	0.679 = 67.8%	-0.339 = 0%	0.661 = 66.1%
HFpow_ARms2_react	0.009	0.238	-0.458 = 0%	0.467 = 46.7%	0.991 = 99.1%
LFpow_ARlog_react	-0.011 = 0	0.008	-0.016 = 0%	0.016 = 1.6%	1 = 100%
HFpow_ARlog_react	-0.420 = 0	-0.042 = 0	0%	0%	100%
LFpow_ARnu_react	0.040	0.261	-0.442 = 0%	0.482 = 48.2%	0.96 = 96%
HFpow_ARnu_react	0.040	0.261	-0.442 = 0%	0.482 = 48.2%	0.96 = 96%
LF_HF_ratio_react	0.068	0.069	-0.002 = 0%	0.07 = 7%	0.932 = 93.2%

Intraclass correlations (ICC) and exploratory Falconer estimates for physiological and subjective stress reactivity measures in the Cyberball test and biofeedback. Falconer estimates (h^2^ = heritability, c^2^ = shared environment, e^2^ = individual environment) should be interpreted with caution due to the limited sample size and instability of several ICC estimates. Values exceeding theoretical ranges (>100%) reflect statistical instability and should not be interpreted as precise estimates of heritability. Negative ICC and c² values were set to zero.

Overall, ICC and Falconer analyses were conducted for exploratory purposes and should be regarded as hypotheses-generating rather than confirmatory.

## Discussion

The present study examined physiological and subjective stress responses during experimentallyinduced social exclusion using the Cyberball paradigm. Overall skin conductance, skin temperature, heart rate variability and subjective stress ratings demonstrated significant phase-related changes, confirmed that the Cyberball task successfully elicited autonomic and subjective stress responses.

As expected, skin conductance increased during exclusion and declined in the recovery phase. This pattern is consistent with previous reports of heightened sympathetic arousal during Cyberball (48–50). However, earlier work also suggested that SC changes may reflect general arousal during gameplay rather than social exclusion per se (19). In the present study, robust phase effects were observed, whereas no significant group differnces emerged. This contrasts with studies showing altered variability in ED populations confronted with body-related stressors (51) or heightened sympathetic response in FGID populations exposed to visceral stressors (52). Skin conductance increased during the cyberball task and declined during recovery. However, despite this clear phase-related pattern, no significant differences emerged between symptom groups, suggesting that the observed autonomic activation reflected a general response to social exclusion rather than a disorder-specific effect within the present sample. The absence of group effects in our sample may also be explained by the relatively small sample size and the use of questionnaire-based symptom groups rather than clinically diagnosed participants.

In addition to skin conductance, skin temperature also showed significant phase effects, decreasing during exclusion and returning to baseline thereafter, in line with prior findings that peripheral temperature drops during social exclusion (25). We also observed phase-dependent changes in skin temperature across the experimental procedure. Although participants with ED-related symptoms tended to show slightly lower temperature values, the overlap of confidence intervals and the absence of significant group effects indicate that these differences should be interpreted cautiously. In line with existing evidence of thermoregulatory alterations in ED (53), this trend is notable but requires further investigations in larger and clinically defined samples.

Heart rate variability analyses also showed phase-dependent changes. SDNN increased during social exclusion, accompanied by significant changes in LF power and HF power expressed by normal units. These findings suggest an adaptive adjustment of autonomic regulation under social stress. An increase in SDNN has previously been linked to greater overall variability and flexibility in cardiovascular regulation (22). While previous studies have reported increased SDNN in ED (54, 55), the present results did not indicate disorder-specific effects.

The low frequency component, reflecting both sympathetic and parasympathetic contributions (55), also increased during exclusion. Earlier studies reported reduced LF in ED, often associated with underweight status (3, 56) or increased LF in anxiety and depression related to FGID (20). However, interpretation of LF-related measures remains challenging because LF power reflects multiple physiological mechanisms and can be influenced by respiratory activity. As respiration was not assessed in the present study, conclusions regarding specific autonomic mechanisms should be cosidered tentative (57).

HF power expressed in normalized units decreased during exclusions, consistent with reduced parasympathetic activity during stress. Previous findings regarding HF activity in ED populations have been heterogeneous (56, 58, 59). In contrast, the LF/HF ratio remained unchanged in the present study and no group differences emerged, suggesting that parasympathetic withdrawal during social exclusion may represent a general stress response rather than a disorder-specific alteration within the present sample.

Given the twin design of the study, autonomic responses were further examined in relation to zygosity and birth order. No significant main effects were found, indicating comparable physiological responses in monozygotic and dizygotic twins. Some interaction effects between group and zygosity were found for RMSSD and high-frequency HRV parameters (HFpow_AR in ms² and log); however, no consistent pattern emerged across indices. These findings should therefore be interpreted cautiously and regarded as exploratory. Previous twin studies have demonstrated that HRV is influenced by both genetic and environmental factors (60, 61). The present findings neither confirm nor contradict these observations but rather highlight the complexity of autonomic stress regulation and the need for replication in larger twin samples.

Subjective stress ratings showed a clear phase effect. Participants reported increased tension during the exclusion phase and decreased ratings during recovery, confirming a clear subjective effect of the Cyberball task. No significant group or zygosity effects were found, although participants with eating disorders-related symptoms tended to report slightly lower stress levels, these differences were not statistically significant.

A significant birth order effect emerged, with second born twins reporting higher stress levels across all phases compared to first-born twins. However, this finding should be interpreted carefully because first-born twins were consistently assessed before second-born twins. Consequently, the observed effect may also partly reflect procedural order effects, such as anticipation, familiarity with the study setting or differences in waiting time, rather than birth-order-related differences per se.

Taken together the Cyberball task induced both physiological (HRV, SC, ST) and subjective (VAS) stress responses, while the largely diagnosis-independent pattern highlights substantial inter-individual variability. This variability raises the question to what extent shared genetic factors versus non-shared environmental influences contribute to stress reactivity under social exclusion.

Against the background of largely diagnosis-independent stress responses to social exclusion, the twin design allowed an exploratory examination of potential genetic and environmental influences on symptom expression and stress reactivity.

Concordance analyses showed higher concordances rates among monozygotic compared to dizygotic twin pairs. Within the FGID symptom group, concordance was higher among monozygotic twins, whereas the EAT group showed generally low concordance and no concordant dizygotic pairs. The EAT+FGID group displayed mixed results, likely reflecting the limited number of twin pairs. As expected, all pairs in the asymptomatic group were concordant regardless zygosity.

Because of the small number of pairs per group, these findings should be interpreted cautiously. Nevertheless, certain tendencies are apparent and align with previous twin research on eating and functional gastrointestinal disorders, both of which have identified a genetic component as well as notable influences of non-shared environmental factors (62, 63). The relatively high rates of discordance in some subgroups further emphasize the role of individual, non-shared environmental influences.

Exploratory ICC and Falconer analyses provided additional insight into potential sources of variability. Falconer estimates approached theoretical boundaries and one estimate exceeded the theoretically interpretable range (>100%). This pattern highlights the instability of these calculations in small samples and further supports interpreting the estimates as exploratory indicators rather than precise measures of genetic and environmental contributions.

For skin conductance and skin temperature, the observed pattern was largely consistent with predominant non-shared environmental influences and only minimal indications of genetic effects. Similarly, most HRV indices showed little evidence of substantial genetic contributions in the present sample.

ICCs quantify the degree of similarity between twins within a pair and indicate the extent to which trait expression is correlated between them (38). These calculations are based on the assumption that monozygotic twins share 100% of their genetic material, whereas dizygotic twins share on average about 50%. Comparisons of within-pair similarities between mono- and dizygotic twins therefor allow estimation of the relative influence of genetic factors (h^2^), shared environment (c^2^) and non-shared environment (e^2^). ICC values close to 1 indicate strong similarity, whereas negative ICCs that occur when within-pair variance exceeds between-pair variance, were interpreted as zero, as described in previous literature (38, 64, 65). Falconer’s formula (37) were then used to derive percentage estimates of h^2^, c^2^ and e^2^. Although these components should theoretically summarize to 100%, small sample sizes and unstable ICC estimates can result in total exceeding 100% or in negative values, which is a known phenomenon in twin research (64, 66). Negative c^2^ values were set to zero, as a negative shared environmental contribution is not theoretically plausible and values above 100% were interpreted as a statistical artefacts rather than true excesses. These values should therefore be regarded as directional indicators that may reflect tendencies rather than precise estimates.

A mixed pattern emerged for HRV parameters. Most indices suggested substantial non-shared environmental influences, whereas MeanRR produced a comparatively large Falconer estimate. Importantly, the estimated heritability for MeanRR exceeded the theoretical range, indicating instability of the calculation and limiting biological interpretation. Consequently, this finding should not be considered evidence of a strong genetic contribution but rather an indication that larger samples and more sophisticated twin models are required.

Overall, the exploratory twin analyses suggest that individual environmental factors may play an important role in physiological stress responses, whereas genetic influences may contribute to certain aspects of subjective stress perception and selected autonomic measures. However, given the limited sample size, the instability of several estimates, and the absence of formal ACE or ADE modelling (33), these findings should be regarded as hypothesis-generating rather than confirmatory.

Future studies using larger twin cohorts and model-based approaches will be necessary to more precisely disentangle genetic and environmental contributions to stress reactivity under social exclusion. Although many of the results did not reach statistical significance, similar non-significant estimates have been used in prior twin studies to indicate possible patterns of heritability and environmental impact (67, 68).

This exploratory twin study investigated physiological and subjective stress response during experimentally induced social exclusion and examined potential genetic and environmental influences on stress reactivity. Across all participants, the cyberball paradigm successfully induced expected autonomic and subjective changes, supporting its utility as a psychosocial stressor.

Despite these robust phase effects, no significant group differences were found between twins with ED-related, FGID-related symptoms, combined symptom profiles and asymptomatic twins. This suggests that stress reactivity to social exclusion may be largely diagnosis-independent in individuals with subsyndromal symptom profiles.

Differences from previous studies that reported disorder-specific alterations may reflect the non-clinical character of the present sample and the limited statistical power associated with the modest sample size. Exploratory analyses of zygosity and birth order showed few consistent effects. While selected interactions involving vagal HRV parameters were observed, these findings were not consistent.

Exploratory analyses of zygosity and birth order indicated few consistent effects. While selected interactions involving vagal HRV parameters were observed, these findings were not consistent across indices and should therefore be interpreted cautiously. Similarly, the observed birth-order effect on subjective stress ratings may reflect procedural order effects, as first-born twins were always assessed before second-born twins.

Twin analyses provided preliminary indications of both genetic and environmental influences. Higher concordance rates among monozygotic compared to dizygotic twins were consistent with a possible contribution of genetic factors to symptom similarity, whereas the substantial discordance observed within several groups highlights the potential importance of non-shared environmental influences. Exploratory ICC and Falconer analyses further suggested that physiological stress responses may be more strongly influenced by individual environmental factors, whereas subjective stress perception may show greater familial aggregation. However, these findings should be regarded as hypothesis-generating given the small sample size and the instability of some estimates.

In summary, the present findings suggest that physiological and subjective responses to social exclusion are shaped by a complex interplay of environmental and potentially genetic influences. The absence of disorder-specific response patterns highlights the importance of individual variability in psychosomatic stress regulation. Future studies should replicate these findings in larger, clinically characterized twin cohorts, apply model-based twin approaches such as ACE or ADE analyses, and incorporate additional physiological measures, including respiration and endocrine markers to improve interpretation of autonomic stress responses. Overall, the study demonstrates the value of twin designs for exploring individual differences in stress reactivity and provides preliminary directions for future psychosomatic research.

## Data Availability

The raw data supporting the conclusions of this article will be made available by the authors, without undue reservation.
